# Supplementary material for: Dummy Template-Based Molecularly Imprinted Membrane Coating for Rapid Analysis of Malachite Green and Its Metabolic Intermediates in Shrimp and Fish
Source: Molecules. 2022 Dec 30;28(1):310. doi: 10.3390/molecules28010310 (PMC9822206; doi:10.3390/molecules28010310)
Supplement: Supplementary file 1 [file molecules-28-00310-s001.zip › molecules-2063837-supplementary.pdf]

Supporting Information

Dummy Template-based Molecularly Imprinted Membrane Coating for Rapid Analysis of Malachite Green and Its Metabolic Intermediates in Shrimp and Fish

Yi Zhang [1,*], Shaofeng Li [1], Yurong Gu [1], Jianying Zhang [2], Zhenfeng Yue [1], Liao Ouyang [1] and Fengjuan Zhao [2]

[1] School of materials and environmental engineering, Shenzhen Polytechnic, Shenzhen 518055, China
[2] Food Inspection & Quarantine Center, Shenzhen Customs, Shenzhen 518045, China
* Correspondence: zhayi@szpt.edu.cn; Tel.: +86-136-3261-5891; Fax: +86-755-2673-1648

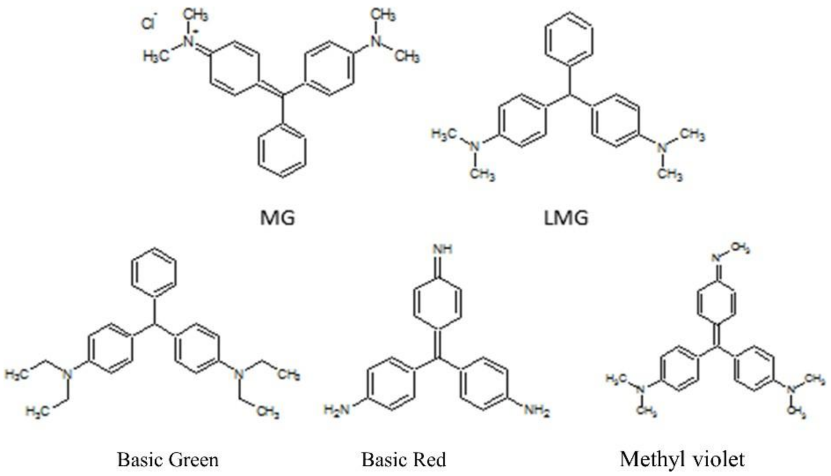

Figure S1. The structure of target and template substitute.

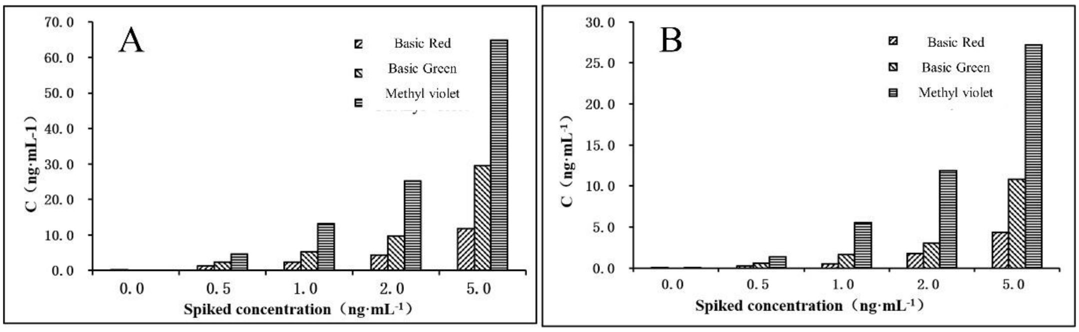

Figure S2. (**A**) Effect of template subsitute on adsorption capacity of MG. (**B**) Effect of templatesub-situte on adsorption capacity of LMG.

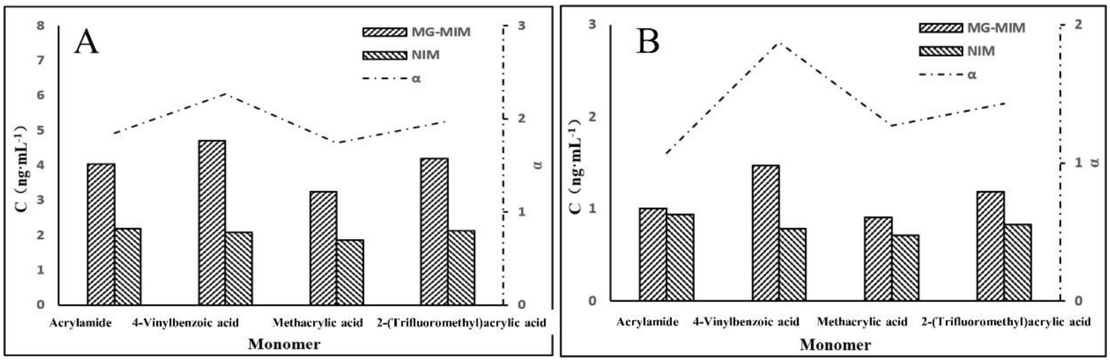

Figure S3. (**A**) Effect of monomer species on adsorption capacity and imprinting factor of MG. (**B**) Effect of monomer species on adsorption capacity and imprinting factor of LMG.

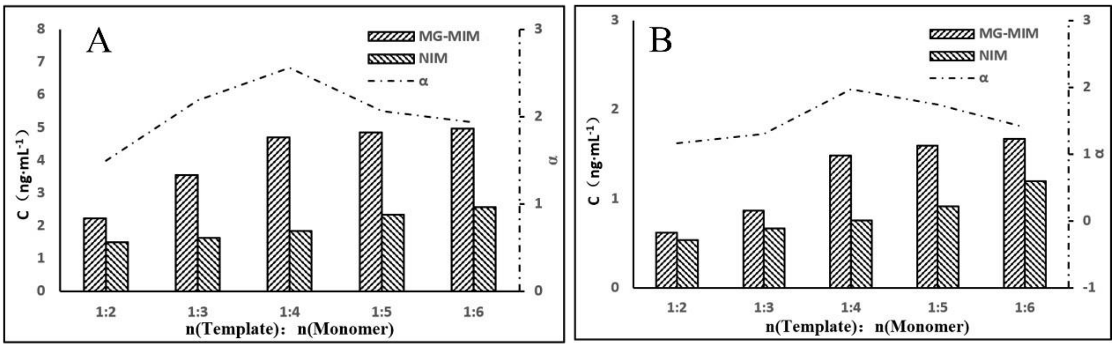

Figure S4. (**A**) Effect of monomer content on adsorption capacity and imprinting factor of MG. (**B**) Effect of monomer content on adsorption capacity and imprinting factor of LMG.

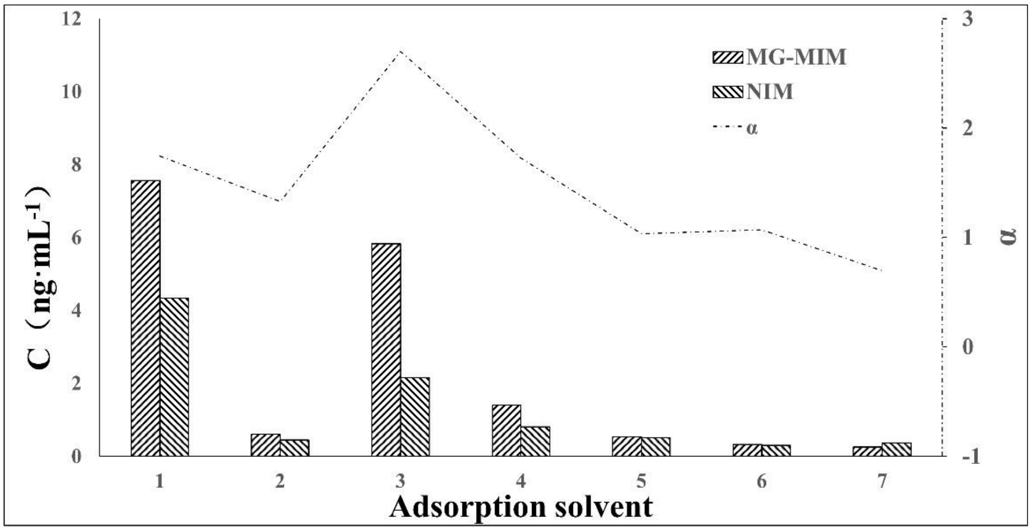

Figure S5. Effect of adsorption solvent on adsorption capacity and imprinting factor of MG. (1) water, (2) ACN, (3) V_{ACN}:V_w = 1:2, (4) V_{ACN}:V_w = 1:1, (5) V_{ACN}:V_w = 3:2, (6) V_{ACN}:V_w = 2:1, (7). V_{ACN}:V_w = 4:1.

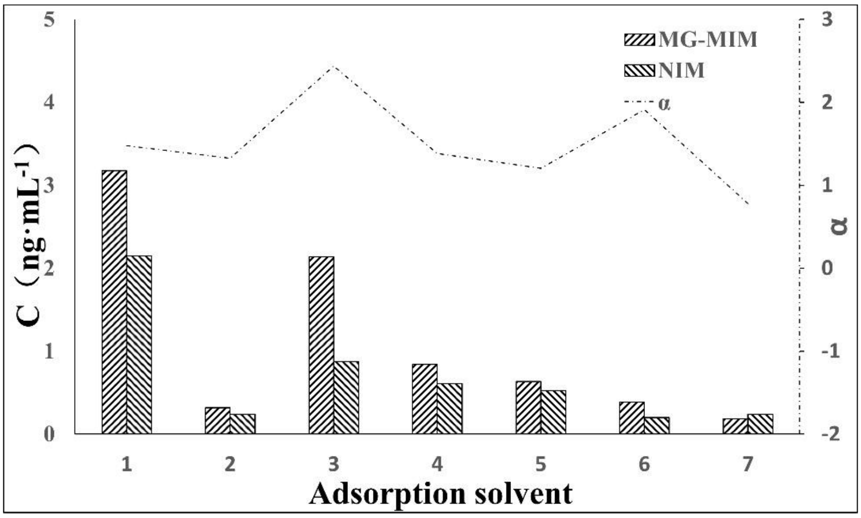

Figure S6. Effect of adsorption solvent on adsorption capacity and imprinting factor of LMG. (1) water, (2) CAN, (3) V_{ACN}:V_w = 1:2, (4) V_{ACN}:V_w = 1:1, (5) V_{ACN}:V_w = 3:2, (6) V_{ACN}:V_w = 2:1, (7) V_{ACN}:V_w = 4:1.

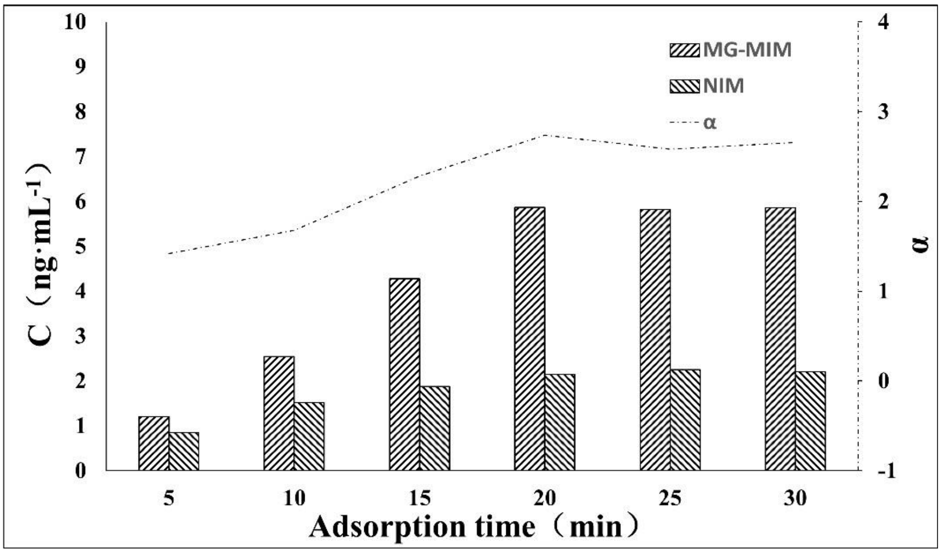

Figure S7. Effect of adsorption time on adsorption capacity and imprinting factor of MG.

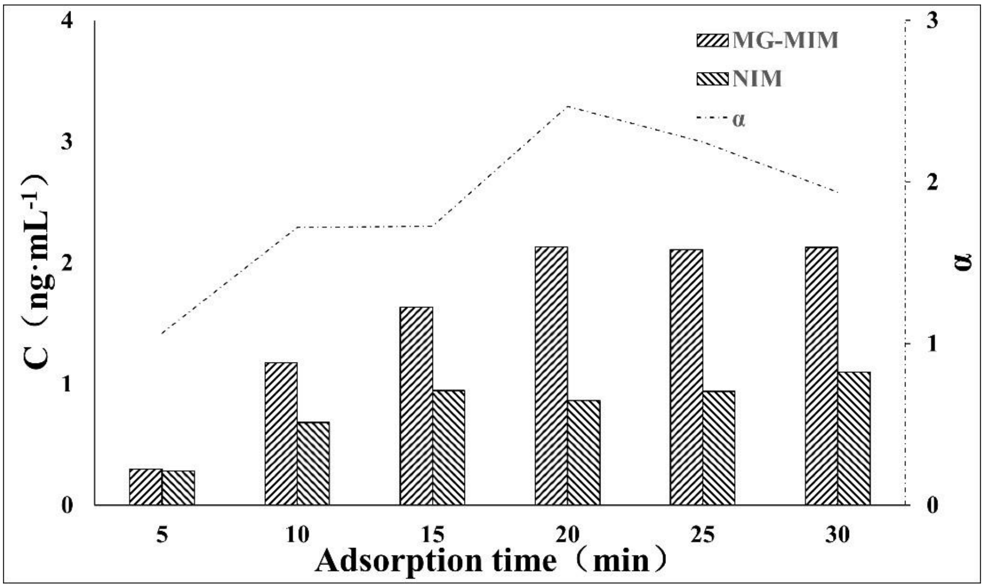

Figure S8. Effect of adsorption time on adsorption capacity and imprinting factor of LMG.

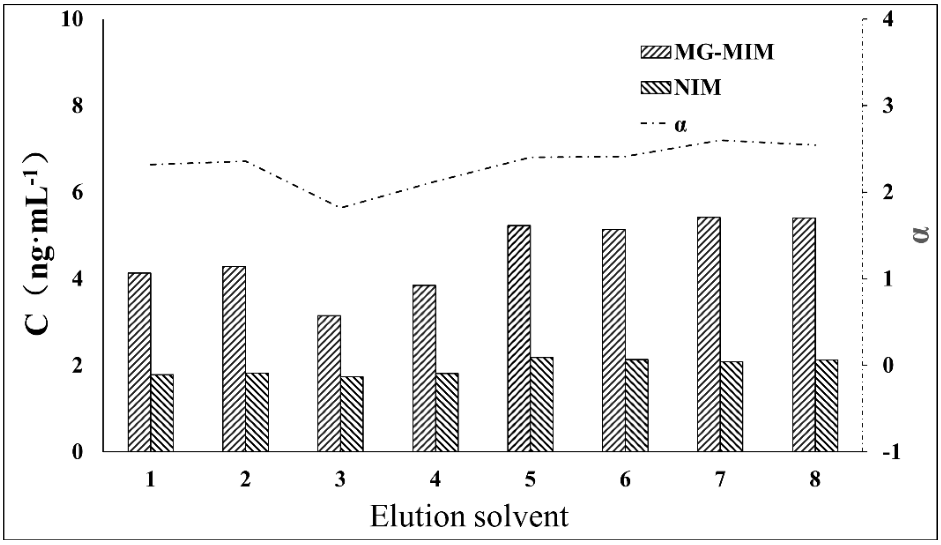

Figure S9. Effect of elution solvent on adsorption capacity and imprinting factor of MG. (1) CAN, (2) acetone, (3) DMSO, (4) DMF, (5) $V_{ammonia}$:V_{ACN} = 1:9, (6) $V_{ammonia}$:$V_{acetone}$ = 1:9, (7) $V_{acetic\ acid}$:V_{ACN} = 1:9 (8), $V_{acetic\ acid}$:$V_{acetone}$ =1:9.

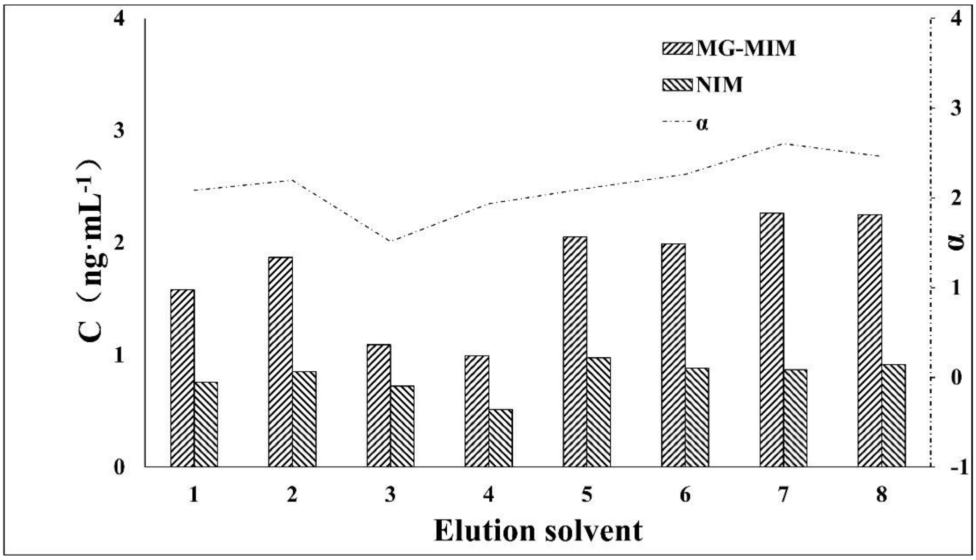

Figure S10. Effect of elution solvent on adsorption capacity and imprinting factor of MG. (1) CAN, (2) acetone, (3) DMSO, (4) DMF, (5) $V_{ammonia}$:V_{ACN} = 1:9, (6) $V_{ammonia}$:$V_{acetone}$ = 1:9, (7) $V_{acetic\ acid}$:V_{ACN} = 1:9 (8), $V_{acetic\ acid}$:$V_{acetone}$ =1:9.

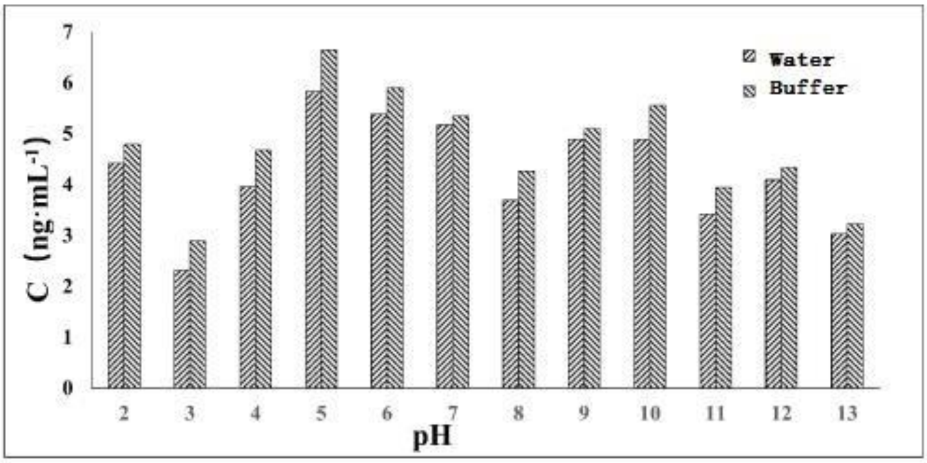

Figure S11. Effect of pH on adsorption capacity of MG.

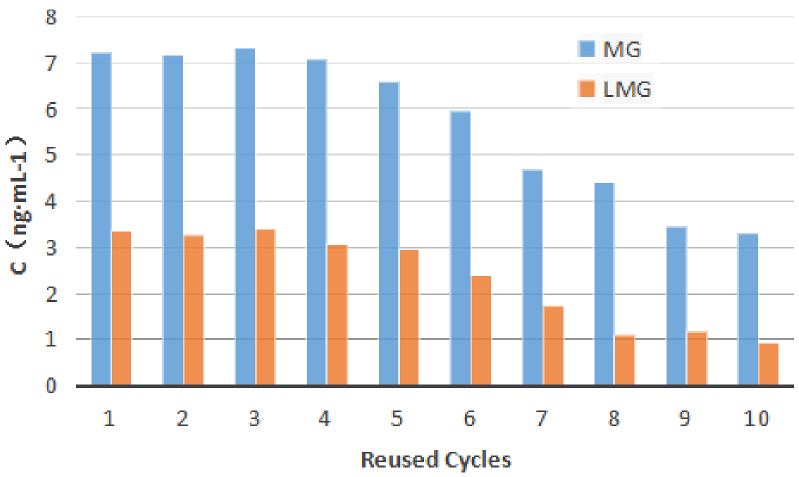

Figure S12. Effect of reused cycles of MG-MIMs.

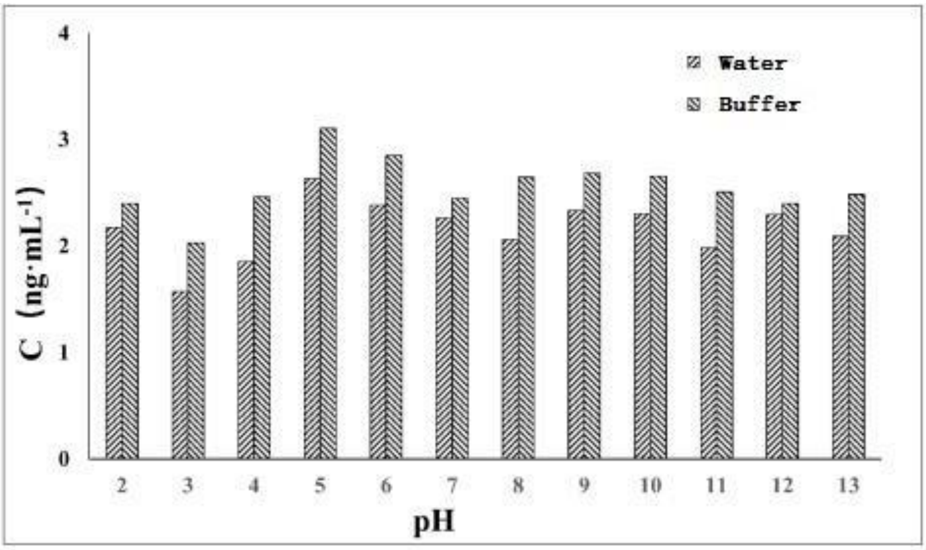

Figure S13. Effect of pH on adsorption capacity of LMG.

Table S1. Effect of ACN on adsorption capacity of MG-MIMs.

t（h）	C(ng·mL^{-1})		RSD（%）	
	MG	LMG	MG	LMG
0	7.29	2.32	\	\
4	7.28	2.25	0.15	3.84
8	7.21	2.34	0.72	0.03
12	7.28	2.28	0.22	2.65
16	7.34	2.36	0.94	0.73
20	7.20	2.40	0.95	2.64
24	7.29	2.40	0.34	2.72

Table S2. Effect of pH on adsorption capacity of MG-MIMs.

pH	C(ng·mL⁻¹)		RSD（%）	
	MG	LMG	MG	LMG
blank	7.23	2.46	\	\
2	7.23	2.26	0.15	8.10
3	7.39	2.56	2.29	4.41
4	7.35	2.31	1.81	6.01
5	7.12	2.28	1.31	7.14
6	7.35	2.42	1.88	1.30
7	7.26	2.38	0.55	2.98
8	7.09	2.66	1.75	7.84
9	7.07	2.57	2.04	4.73
10	7.04	2.65	2.46	7.55
11	7.27	2.45	0.79	0.07
12	7.34	2.50	1.63	2.29
13	7.08	2.33	1.90	4.89
